# Two Cases of Conservative Treatment for Fractured Trapezial Tubercles

**DOI:** 10.7759/cureus.113969

**Published:** 2026-08-04

**Authors:** Toshiaki Kiribayashi, Taiki Ueno, Hayato Seki, Kazuki Shimoonoda

**Affiliations:** 1 Department of Judotherapy and Sports Medicine, Faculty of Health Sciences, SBC Tokyo Medical University, Urayasu, JPN; 2 Department of Orthopaedic Surgery, SBC Tokyo Medical University, Orthopedic Clinic Kamiaoki Branch, Kawaguchi, JPN; 3 Center for Fundamental Education, Faculty of Health Sciences, SBC Tokyo Medical University, Urayasu, JPN

**Keywords:** conservative fracture treatment, fall injury, nonunion fracture, thumb spica cast, trapezial tubercle fracture

## Abstract

Among non-scaphoid carpal fractures, trapezium fractures are uncommon but rank as the third or fourth most frequent. A few international studies suggest that these fractures represent 1.5-3% of fractures of the hand. There are very few reports on the conservative treatment of patients with this type of injury owing to various complications. Herein, we present two cases of trapezial tubercle fractures managed conservatively with thumb spica casting. In Case 1, a 52-year-old male lost control and fell from his motorcycle, resulting in an injury to his right hand. On the following day, he presented to the Kamiaoki Clinic of Orthopedic Surgery, SBC Tokyo Medical University, where computed tomography (CT) revealed a non-displaced fracture of the trapezial tubercle. The patient was fitted with a thumb spica cast and discharged. A follow-up CT obtained six weeks after the injury revealed callus formation without displacement, and the patient's clinical course was uneventful. In Case 2, an 88-year-old woman experienced a fall and injury to the right hand. On the following day, she presented to the Kamiaoki Clinic of Orthopedic Surgery, SBC Tokyo Medical University. Plain radiographs demonstrated a fracture line suggestive of a trapezial injury, and CT confirmed a non-displaced fracture of the trapezial tubercle. The patient was fitted with a thumb spica cast and discharged. Although a CT scan obtained four weeks after the injury showed no obvious callus formation, there was no exacerbation of the fracture; therefore, the spica cast was removed. At the final follow-up, nine weeks after the initial injury, the patient had no difficulty performing activities of daily living. Although previous studies have generally reported satisfactory outcomes following conservative treatment of trapezial tubercle fractures, non-union has also been described and is sometimes considered an indication for surgical intervention. In Case 2, although bony union was not radiographically confirmed, a satisfactory functional outcome was achieved. Given the patient’s advanced age and the higher risk of joint stiffness, the clinical decision to prioritize early mobilization over radiographic union may have facilitated the preservation of her activities of daily living. While Case 1 and Case 2 differ in age and fracture type, Case 2 illustrates that in certain elderly patients with minimally displaced trapezial tubercle fractures, acceptable function can be maintained even without definitive bony union.

## Introduction

Among non-scaphoid carpal fractures, trapezium fractures are uncommon but rank as the third or fourth most frequent. Previous studies suggest that these fractures account for 1.5-3% of hand fractures [1-3], and they are frequently overlooked at the initial diagnosis [4]. The trapezium articulates with the scaphoid, trapezoid, and the base of the first and second metacarpals, and its palmar tubercle serves as the origin of the thenar musculature and the attachment site of the transverse carpal ligament, making it essential for thumb opposition and grip. Trapezial fractures are broadly classified into body fractures and ridge (tubercle) fractures, with the latter further subdivided according to whether the fracture occurs at the base or the tip of the ridge. Body fractures typically result from high-energy trauma or a direct blow to the thenar eminence, whereas ridge fractures most commonly result from a fall onto an outstretched hand. Reported complications include non-union, chronic wrist pain, and weakness of pinch and grip strength, and, in body fracture cases, instability or degenerative change of the first carpometacarpal joint. Although the trapezium plays an essential role in thumb opposition, reports on conservative management remain limited owing to concerns regarding complications [5]. We describe two cases of trapezial tubercle fractures managed conservatively with thumb spica casting, with acceptable clinical outcomes at short-term follow-up.

## Case presentation

Case 1

**Figure 1:**
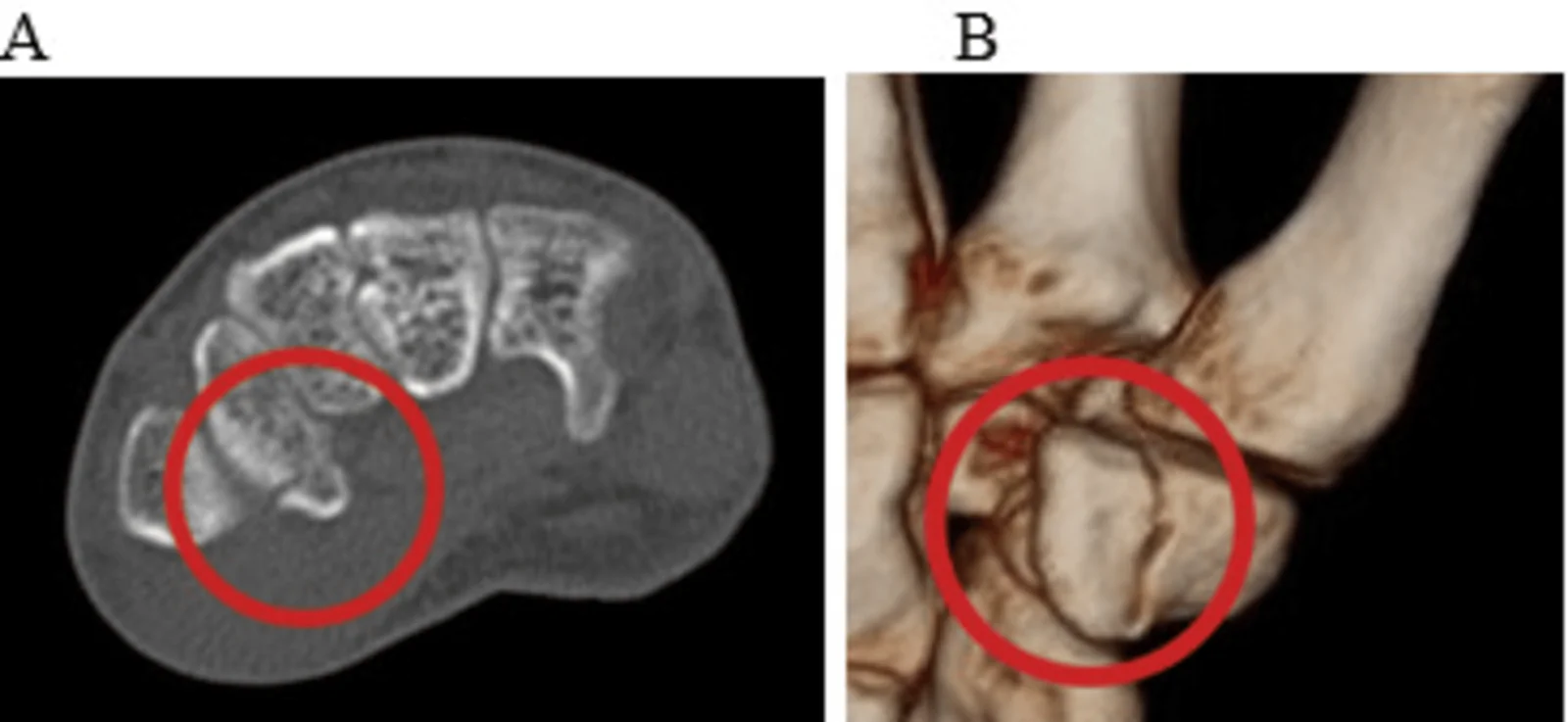
Case 1: Initial computed tomography (CT) findings. Axial (A) and three-dimensional (B) CT images showing a non-displaced fracture of the trapezial tubercle (red circles)．

**Figure 2:**
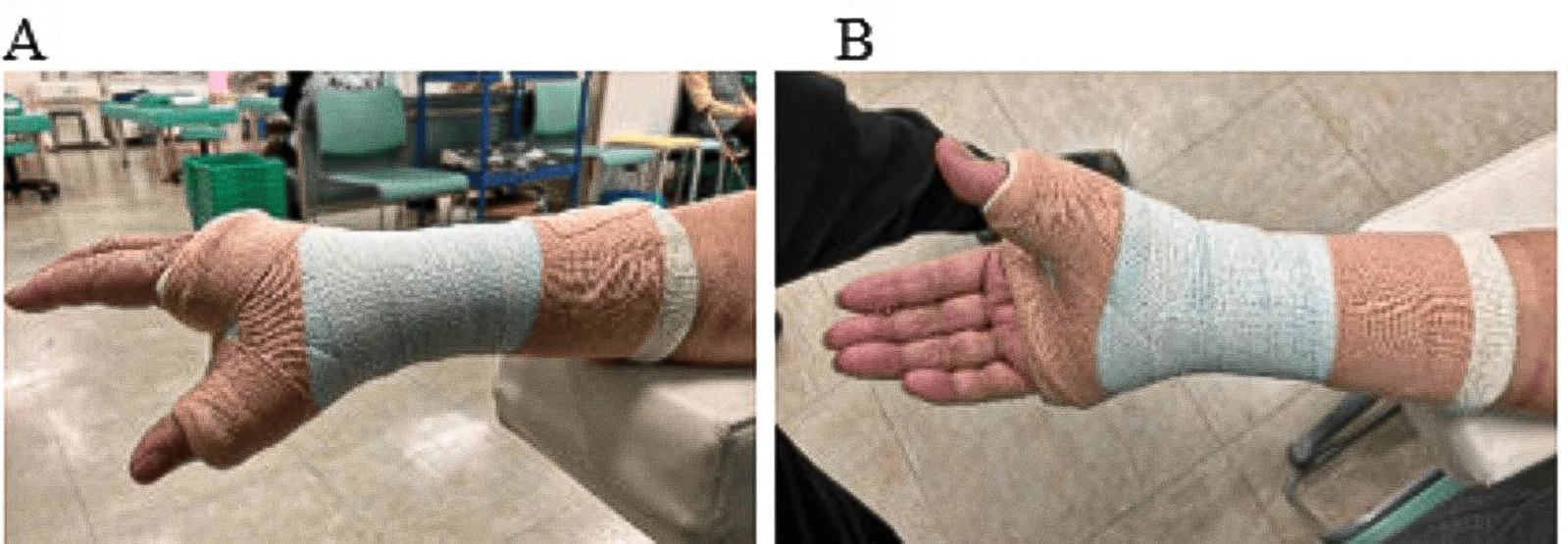
Initial fixation. Photographs of the radial (A) and palmar (B) sides of the wrist. A thumb spica cast was fitted from the center of the forearm to the area immediately before the interphalangeal joint of the thumb. The same fixation method was used in both cases.

**Figure 3:**
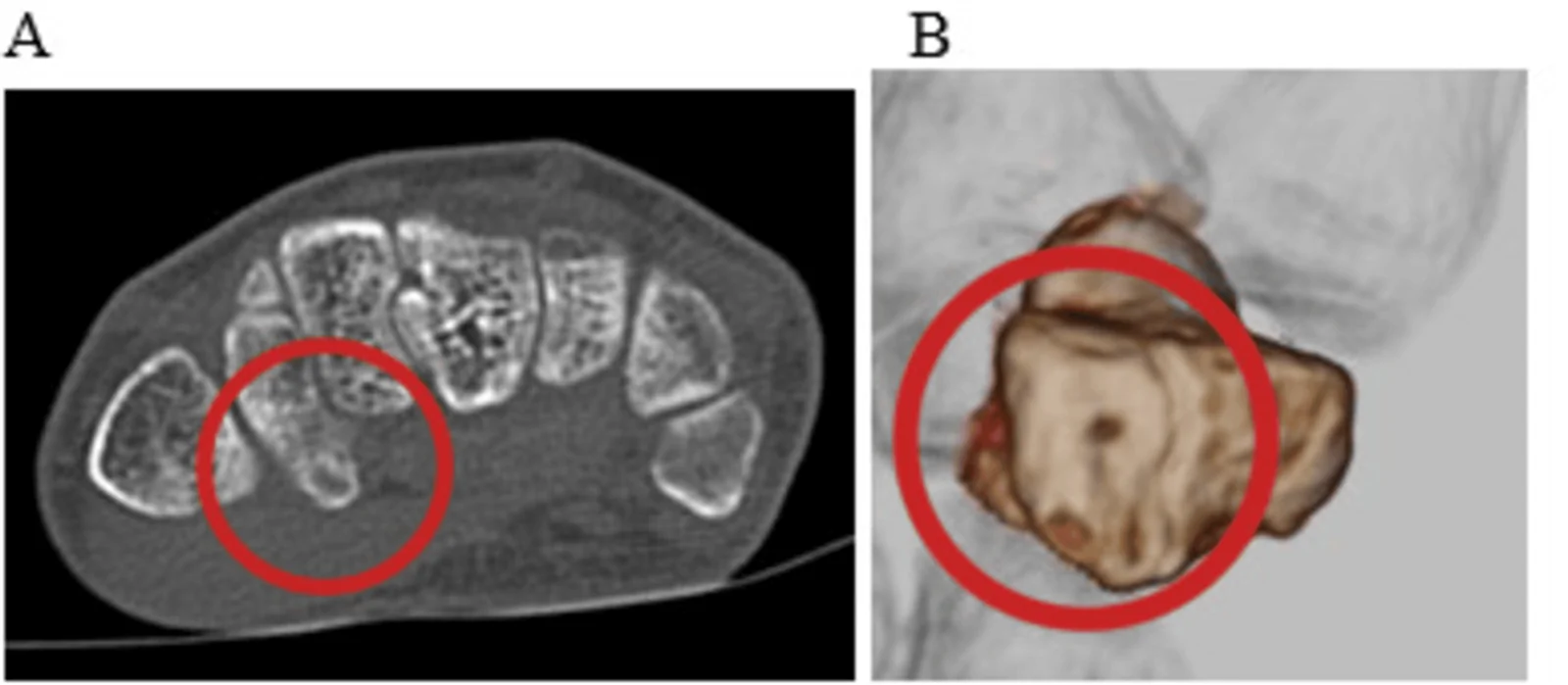
Case 1: CT images obtained six weeks post-injury. Axial (A) and three-dimensional (B) CT images showing callus formation with no exacerbation of the fracture (red circles).

A 52-year-old male lost control and fell from his motorcycle, resulting in an injury to his right hand. On the following day, he presented to the Kamiaoki Clinic of Orthopedic Surgery at SBC Tokyo Medical University. On arrival, mild swelling and pronounced tenderness were noted on the radial side of his wrist. Computed tomography (CT) revealed a non-displaced fracture of the trapezial tubercle (Figure 1), and the patient was fitted with a thumb spica cast (Figure 2). At a follow-up appointment six weeks after injury, CT demonstrated callus formation consistent with bony union (Figure 3), with no evidence of displacement at the fracture site. After the cast was removed, the patient still had some range of motion (ROM) limitations; therefore, he underwent electrical stimulation therapy and ROM training. The patient continued to report mild pain on full extension; however, full wrist extension (dorsiflexion) was achieved, matching the contralateral side, and wrist ROM was symmetrical with the contralateral side on clinical examination, although detailed goniometric measurements were not recorded, and there was no impairment in activities of daily living.

Case 2

**Figure 4:**
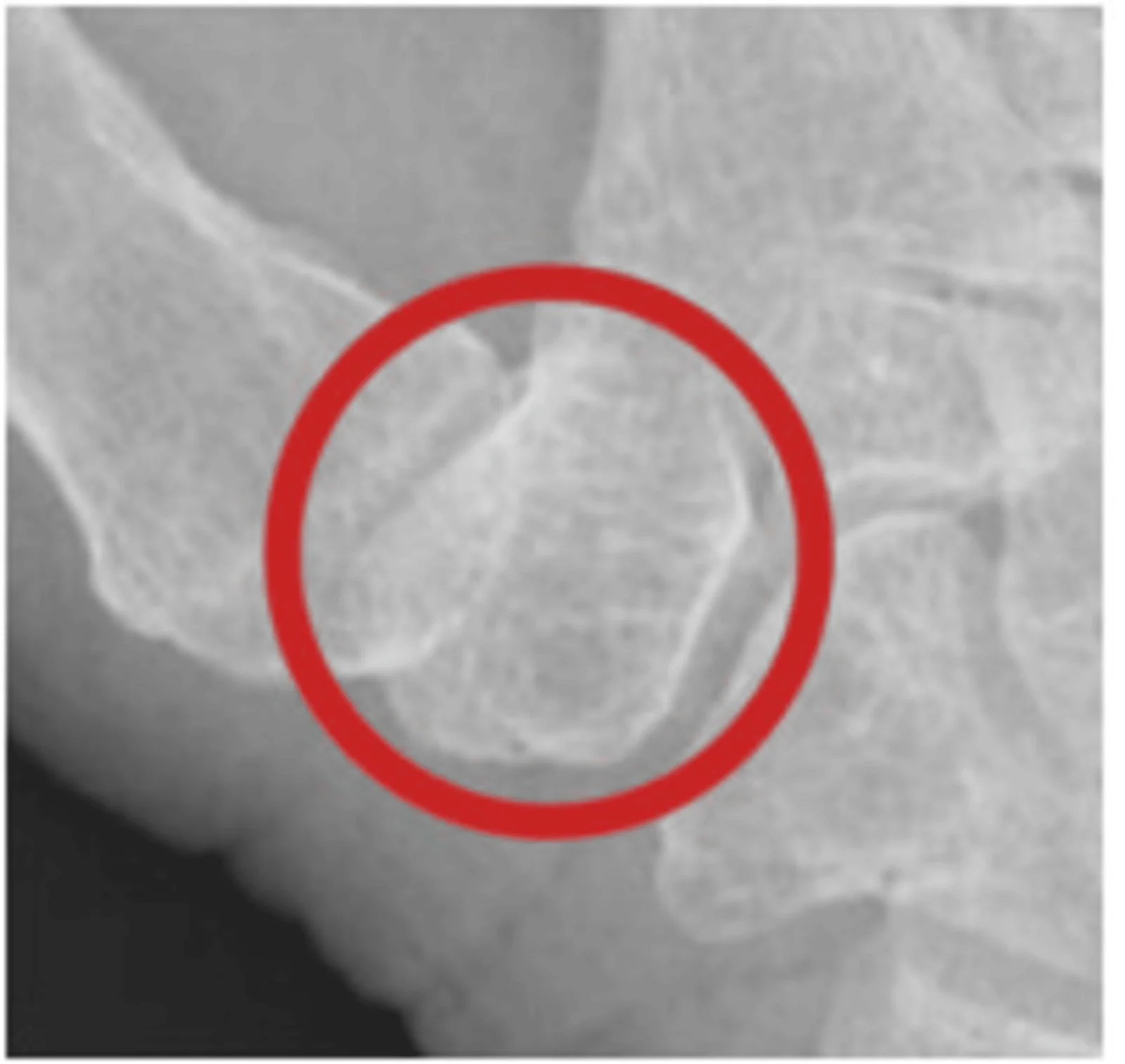
Case 2: Radiograph of the right wrist showing a fracture line suggestive of a trapezium fracture, consistent with the location of tenderness (red circle).

**Figure 5:**
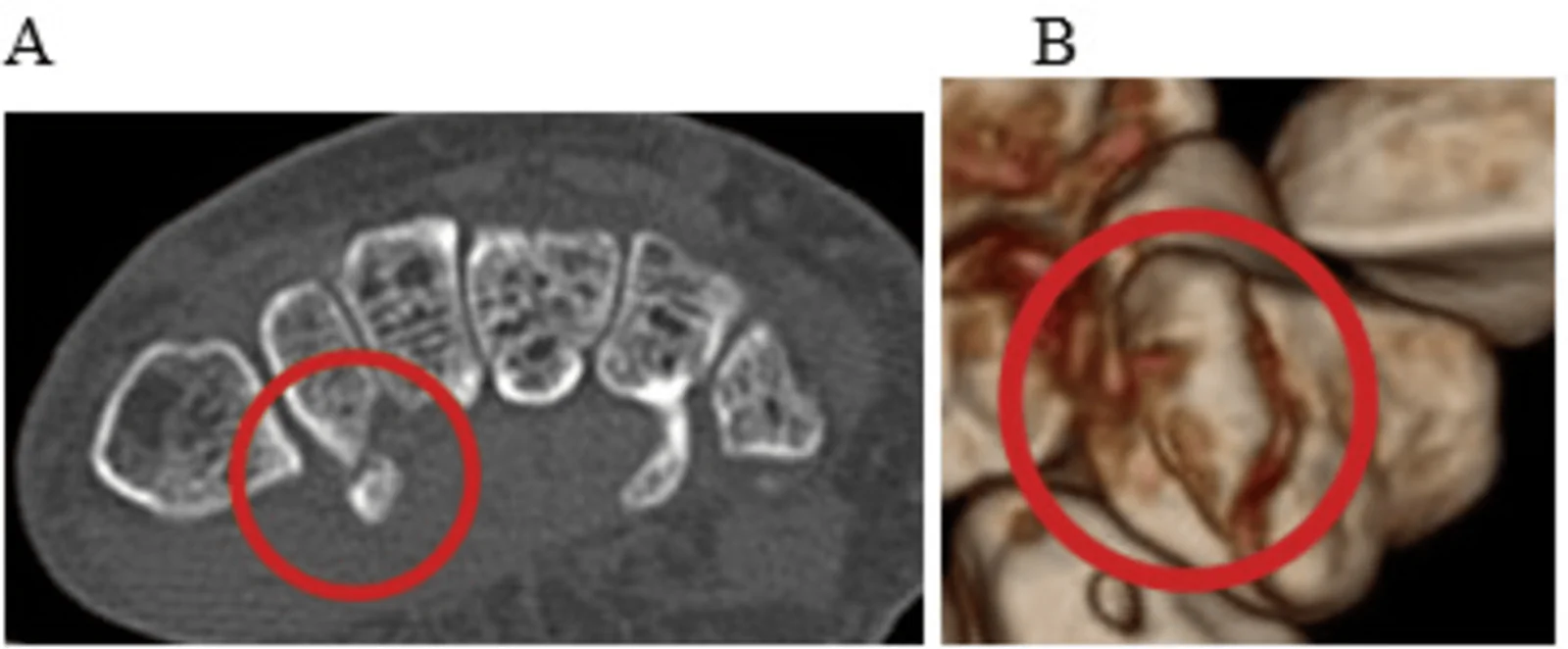
Case 2: Initial CT findings. Axial (A) and three-dimensional (B) CT images showing a fracture line in the trapezial tubercle (red circles).

**Figure 6:**
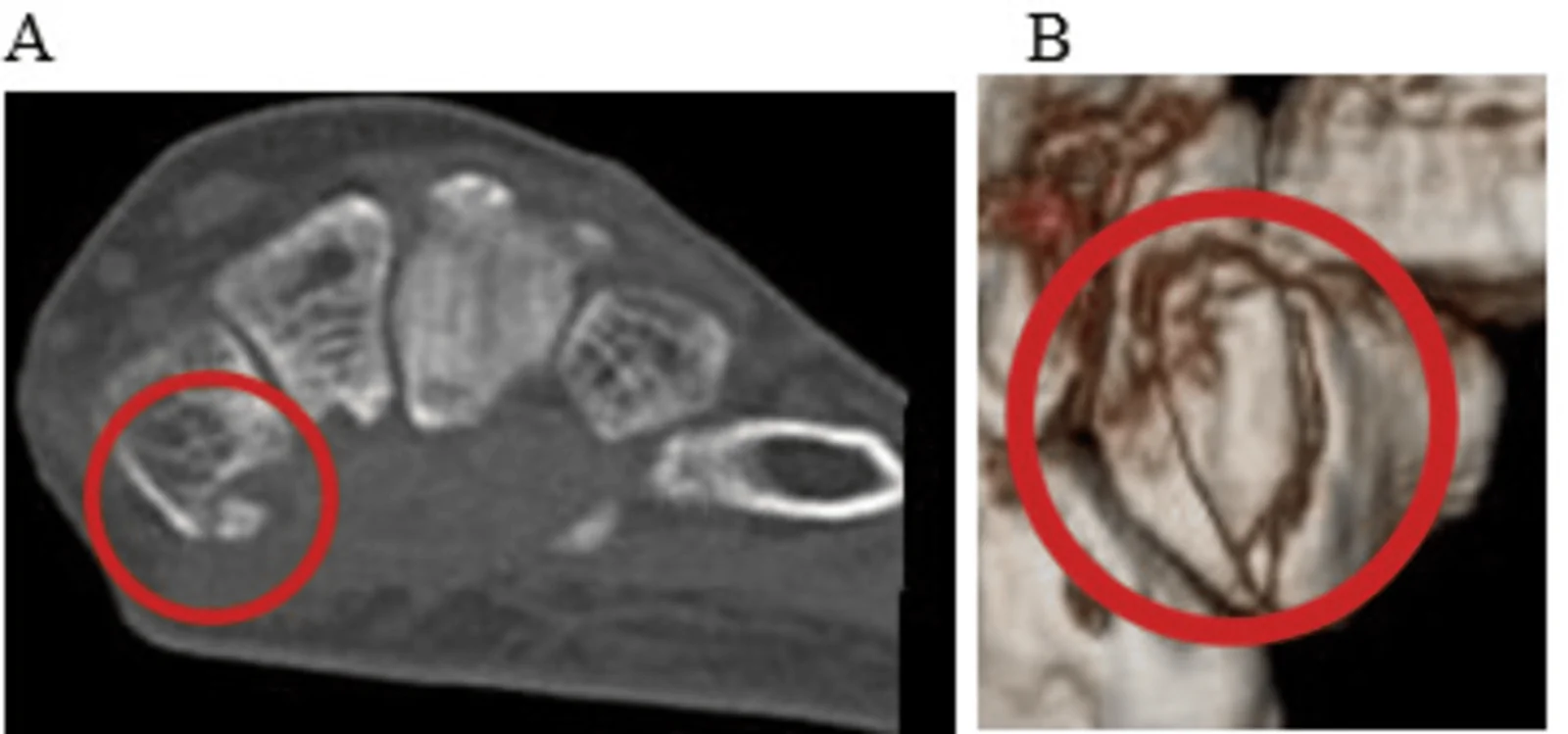
Case 2: CT images obtained four weeks post-injury. Axial (A) and three-dimensional (B) CT images showing an absence of both fracture exacerbation and callus formation (red circles).

**Figure 7:**
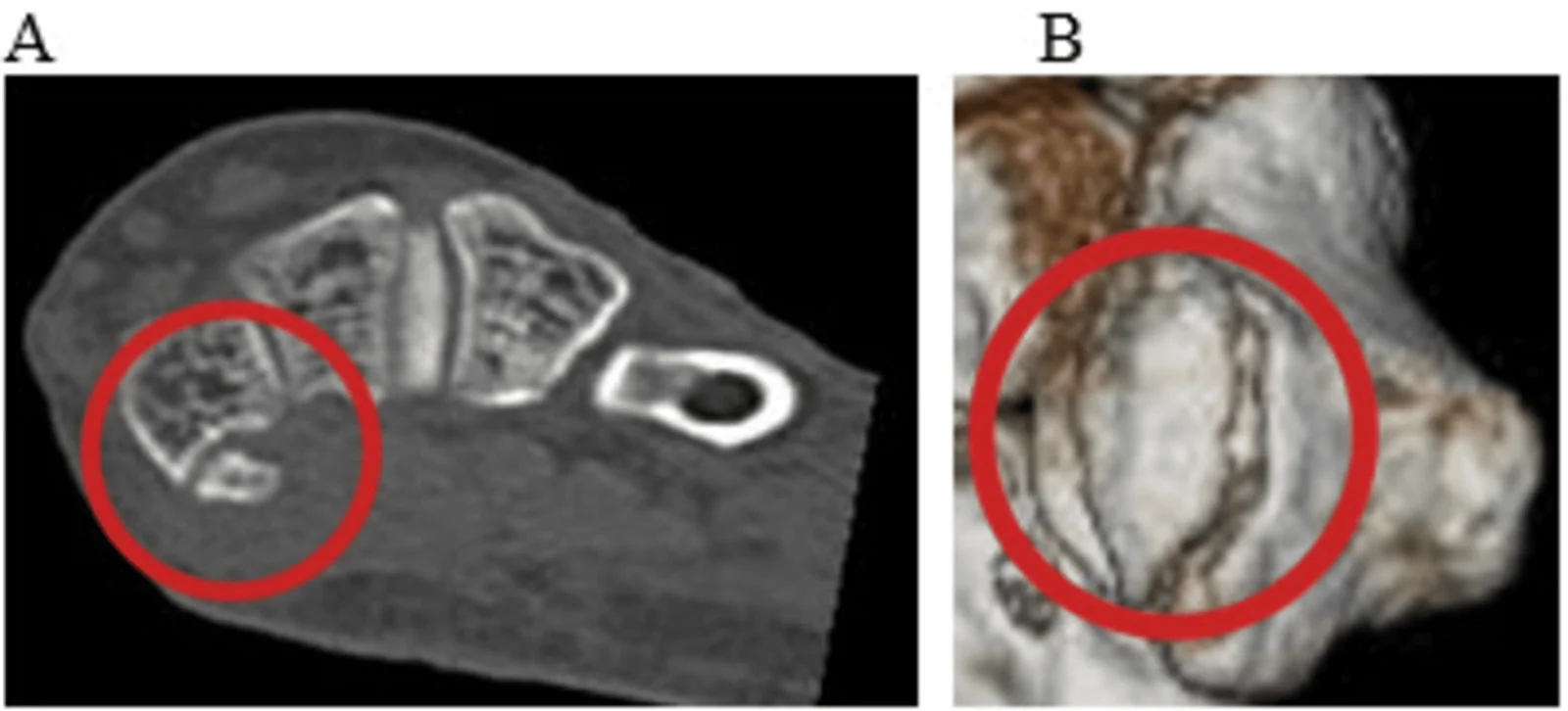
Case 2: Final CT images obtained nine weeks post-injury. Axial (A) and three-dimensional (B) CT images showing similar results to those at four weeks – both fracture exacerbation and callus formation were absent (red circles).

An 88-year-old right-handed female with a history of hypertension and diabetes fell and injured her dominant right hand. On the following day, she presented to the Kamiaoki Clinic of Orthopedic Surgery at SBC Tokyo Medical University. On presentation, significant swelling and tenderness were observed from the tubercle of the scaphoid bone to the carpometacarpal joint of the thumb. Radiography revealed a fracture line suggestive of a trapezial injury (Figure 4), and CT confirmed a non-displaced fracture of the trapezial tubercle (Figure 5). A thumb spica cast was applied prior to discharge (Figure 2). CT images obtained four weeks after the injury showed no obvious callus formation, although the fracture remained non-displaced (Figure 6). Given the patient's advanced age and the risk of ROM restriction with prolonged immobilization, the cast was removed at that time. On the final CT obtained nine weeks after injury, bony union was not clearly demonstrated (Figure 7); however, the patient reported no pain or tenderness during movement. Goniometric measurements of wrist and thumb ROM were obtained at cast removal (four weeks post-injury) and at the final follow-up (nine weeks post-injury). During cast removal, wrist flexion/extension/radial deviation/ulnar deviation were 50°/50°/25°/40° on the affected side versus 55°/50°/25°/40° on the contralateral side; thumb radial abduction/ulnar adduction/palmar abduction/palmar adduction were 35°/0°/50°/0° versus 60°/0°/55°/0°; and thumb MCP flexion/extension and IP flexion/extension were 35°/10°/70°/5° versus 40°/10°/75°/5°. At the final follow-up, wrist flexion/extension/radial deviation/ulnar deviation improved to 65°/50°/25°/40° on the affected side versus 60°/50°/25°/50° on the contralateral side; thumb radial abduction/ulnar adduction/palmar abduction/palmar adduction were 45°/0°/50°/0° versus 50°/0°/50°/0°; and thumb MCP flexion/extension and IP flexion/extension were 35°/10°/70°/5° versus 35°/10°/70°/5°, essentially matching the contralateral side except for mild residual restriction in wrist ulnar deviation (40° vs. 50°) and thumb radial abduction (45° vs. 50°). Grip strength, measured with a dynamometer, was 19.1 kg on the affected (dominant) side versus 18.5 kg on the contralateral side, that is, slightly greater on the affected side, and full grip was possible. While a standardized functional score (e.g., DASH) was not obtained, these quantified goniometric and grip strength measurements, together with the patient's reported absence of impairment in activities of daily living, indicate a satisfactory functional recovery despite the lack of definitive radiographic bony union. 

## Discussion

As noted above, trapezium fractures are uncommon but clinically important given their role in thumb function. Cordrey et al. [6] stated that the main symptoms of trapezium fractures were weakness and restricted movement of the thumb and that swelling and pain were generally mild, indicating that a careful diagnosis is required. However, our cases exhibited more acute clinical presentations; Case 1 presented with pronounced tenderness, and Case 2 demonstrated significant swelling and tenderness. These findings contrast with the typically mild symptoms described by Cordrey et al. [6], suggesting that clinical presentations can vary in severity and that acute inflammatory signs do not necessarily rule out this injury.

The outlines of the trapezium and trapezoid overlap on posteroanterior, lateral, and oblique radiographs of the wrist, making trapezium fractures easy to overlook. Therefore, careful consideration of the mechanism of injury and the patient’s clinical findings is crucial when a fracture is suspected. In such cases, where the definitive fracture line is unclear, but the location of tenderness is consistent with trapezial injury, we believe that CT is the best imaging modality for assessing the anatomy.

Cordrey et al. [6] first classified trapezium fractures into three types: ridge-type fractures involving the tubercle, vertical-type fractures with fracture lines perpendicular to the body, and comminuted fractures. Walker et al. [7] further classified trapezium fractures into five types, adding types IIa and IIb fractures classified as those accompanied by Bennett fractures. Whereas Cordrey et al.'s [6] system was based primarily on fracture morphology, Walker et al.'s [7] classification incorporated concomitant carpometacarpal fracture-dislocation, reflecting a shift toward classifications with greater surgical relevance; however, neither system further subdivides ridge-type (tubercle) fractures by the level of injury, a distinction addressed later in this section and directly relevant to the present cases.

In their review of the literature on trapezium fractures, Pointu et al. [8] identified 88 cases, which included 34 patients evaluated directly by the authors. The fracture patterns were categorized into vertical, horizontal, oblique, and comminuted types. The mechanisms of injury for trapezial fractures include both direct and indirect external forces, and it is assumed that the mechanisms differ depending on the type of fracture. High-energy trauma, including motor vehicle accidents and contact sports, frequently accounts for fractures of the trapezial body [9,10]. The instability associated with these fractures often predisposes patients to dislocation of the first carpometacarpal joint [11]. Concurrent injuries to other carpometacarpal structures, such as the scaphoid or the base of the first metacarpal, are frequently observed alongside these fractures [10,12,13]. A fall onto an outstretched hand is the primary mechanism driving trapezial ridge fractures, which manifest either as direct trauma to the ridge (Type I) or as a ligamentous avulsion (Type II) triggered by tension on the transverse carpal ligament (TCL) [14]. The trapezial tubercle is also referred to as the ridge [14]. Anatomically, Type I fractures are located at the base of the ridge, while Type II fractures involve the tip [15,16]. The literature suggests that Type I ridge fractures generally achieve good clinical outcomes with conservative management, such as thumb spica immobilization for four to six weeks [15,17]. Both cases in this report involved fractures of the trapezial tubercle; Case 1 was classified as Type I and achieved clear bony union after six weeks of casting. By contrast, Case 2 was a Type II fracture, which is reported to carry a higher risk of non-union [15]. In symptomatic patients with Type II fractures, early surgical excision of the fragment is often considered to ensure favorable functional outcomes and avoid the prolonged morbidity associated with a painful nonunion. However, Case 2 illustrates that even in a Type II fracture, a satisfactory functional result can be achieved without surgery by avoiding prolonged immobilization, particularly in elderly patients where preserving immediate activities of daily living is a priority. Gibney et al. [14] demonstrated that fractures of the trapezium are the most frequent carpal fractures missed on initial radiography. In addition, these injuries have been highlighted as an underdiagnosed source of post-traumatic wrist pain that frequently presents with clinical features similar to those of scaphoid fractures. Beekhuizen et al. [18] state that fractures of the trapezium are rare and often unrecognized lesions; therefore, surgeons must pay particular attention to avoid missing these injuries in the emergency department. The initial evaluation should include plain radiography; however, plain films have limited sensitivity for trapezial tubercle fractures because the fracture line often overlaps with adjacent carpal structures on standard posteroanterior, lateral, and oblique views, and a specific carpal tunnel view may be required to visualize the tubercle in profile. If no fracture is identified on radiography but there remains a high suspicion of carpal fracture, an additional CT scan should be performed. Taken together, these two reports [14,18] consistently point to radiographic underdiagnosis as the principal barrier to timely treatment, rather than any inherent difficulty in clinical detection, underscoring that liberal use of CT - rather than reliance on plain radiography alone - is warranted whenever trapezial injury is clinically suspected, as was our diagnostic approach in both cases. Clinically, reproducible tenderness over the trapezial tubercle and pain elicited by resisted palmar flexion or opposition of the thumb-reflecting tension transmitted through the thenar musculature and transverse carpal ligament attaching to the tubercle-may raise suspicion for this fracture prior to imaging, although these specific provocative maneuvers were not formally documented in the present cases and their diagnostic value warrants further evaluation. 

In Case 1, a clear bone union was observed on the follow-up CT images, and the patient experienced no impairment in activities of daily living. However, in Case 2, although bone union was not clearly observed on the final CT images, the patient experienced no impairment in performing activities of daily living. In both cases, fracture displacement was minimal, and preservation of activities of daily living was observed despite differing durations of immobilization; however, whether earlier cast removal specifically contributed to this outcome in Case 2 cannot be determined from a single case, given the multiple factors involved (including minimal fracture displacement and the patient's baseline function), and this remains a hypothesis for future study rather than a confirmed finding. Anatomically, Type I fractures are located at the base of the ridge, while Type II fractures involve the tip [15,16]. Both cases in this report involved fractures of the trapezial tubercle; Case 1 was classified as Type I and Case 2 as Type II. While Type II fractures are associated with a higher risk of non-union [15], given the small, ligament-tethered nature of the avulsed fragment, non-union should be regarded as an expected outcome of this specific injury pattern rather than a failure of treatment. As such, management decisions should be guided by clinical symptoms rather than radiographic union. Asymptomatic non-unions, as seen in Case 2, can be managed expectantly, especially when avoiding prolonged immobilization is a priority to preserve activities of daily living. Surgical intervention, such as early excision, should be reserved for patients who remain symptomatic, as it typically provides definitive and good functional outcomes.

For patients managed conservatively, however, longer-term observation is warranted for several reasons. First, radiographic non-union, as seen in Case 2, does not preclude the possibility of delayed union or fibrous union providing sufficient stability; however, whether this stability is maintained over months to years remains unknown. Second, persistent non-union of the trapezial tubercle could theoretically predispose patients to late complications such as chronic instability of the thumb carpometacarpal joint, degenerative change at the scaphotrapeziotrapezoid (STT) joint, or delayed-onset pain with increased mechanical loading. Third, the follow-up periods in this report (six and nine weeks) are too short to exclude late-onset symptoms or functional decline, particularly in the elderly patient in Case 2, whose bone healing capacity may differ from that of younger patients. Longer-term follow-up, ideally extending to at least six months to one year with repeat imaging and functional assessment, would be necessary to confirm whether the favorable short-term outcomes observed here are sustained, and to determine whether asymptomatic non-union in Type II trapezial ridge fractures carries a risk of subsequent complications.

## Conclusions

We presented two cases of trapezial tubercle fracture managed conservatively with thumb spica casting. In Case 1, CT images obtained six weeks post-injury showed clear bone union, and the patient experienced no impairment in activities of daily living. In Case 2, although bony union was not radiographically confirmed at nine weeks, the patient had recovered her baseline activities of daily living. However, it must be acknowledged that a nine-week follow-up is insufficient to rule out long-term complications associated with trapezial non-union, such as post-traumatic osteoarthritis, late-onset pain, or flexor carpi radialis tendinopathy. Given the patient's advanced age and relatively low functional demands, her current lack of symptoms may not fully represent the long-term durability of this clinical outcome. The decision to prioritize early mobilization was made to prevent debilitating joint stiffness, but the resulting radiographic non-union remains a significant concern. Therefore, the conclusion that conservative management with early mobilization is effective for Type II fractures must be interpreted with caution. This study is significantly limited by its short follow-up period, and further longitudinal research is essential to determine if such asymptomatic non-unions remain stable or eventually lead to functional decline over several years. In both cases, fracture displacement was minimal; however, whether the avoidance of prolonged immobilization causally contributed to the preservation of activities of daily living cannot be determined from these two cases alone and remains a hypothesis for future study. As discussed above, longer-term follow-up is required to confirm whether the favorable short-term functional outcomes observed in these cases, particularly the asymptomatic non-union in Case 2, are sustained over time.
